# Machine learning-based stratification of mild cognitive impairment in Parkinson’s disease: a multicenter cross-sectional analysis

**DOI:** 10.1186/s12911-025-03215-0

**Published:** 2025-10-15

**Authors:** Yanfang Liu, Meiling Chen, Peng Chen, Xiaohui Lin, Sangsang Chen, Chaoning Liu, Donghui Wang, Hongxing Deng, Qinghua Li, Yuan Wu

**Affiliations:** 1 Department of Neurology, The First Affiliated Hospital of Guangxi Medical University, No. 6 Shuangyong Road, Nanning, Guangxi 530021 China; 2 Department of Presbyatrics, The Affiliated Hospital of Guilin Medical University, No. 15 Lequn Road, Guilin, Guangxi 541002 China; 3 Department of Neurology, The Affiliated Hospital of Guilin Medical University, No. 15 Lequn Road, Guilin, Guangxi 541002 China; 4 Guangxi Clinical Research Center for Neurological Diseases, The Affiliated Hospital of Guilin Medical University, Guilin, Guangxi China

**Keywords:** Parkinson’s disease, Mild cognitive impairment, Machine-learning model, Nomogram, Shapley additive explanations, Montreal cognitive assessment

## Abstract

**Background:**

Cognitive impairment is a prominent non-motor manifestation of Parkinson’s disease (PD) and is associated with reduced quality of life, increased mortality, and higher healthcare utilization. We aimed to develop and externally validate a machine-learning model, trained on Montreal Cognitive Assessment (MoCA)—based Movement Disorder Society (MDS) Level I labels, that estimates the contemporaneous probability of mild cognitive impairment in PD (PD-MCI) from routinely collected clinical variables, enabling clinicians to prioritize MoCA-normal patients with higher model-estimated probability for MDS Level II neuropsychological evaluation and closer follow-up.

**Methods:**

We analyzed 799 participants with PD from the Parkinson’s Progression Markers Initiative (PPMI), randomly assigning them to training (*n* = 559) and internal validation (*n* = 240) cohorts. An independent external cohort comprised 70 consecutive patients recruited at The Affiliated Hospital of Guilin Medical University between February 2024 and March 2025. The reference outcome was MoCA-based PD-MCI (21–25) versus cognitively normal PD (26–30). Candidate predictors were screened by LASSO (1-SE criterion). To handle class imbalance, SMOTE was applied only during model fitting; both validation cohorts retained native class distributions. Five machine-learning models (logistic regression [LR], support vector machine, XGBoost, neural network, LightGBM) were evaluated on non-resampled data for discrimination (area under the receiver operating characteristic curve, AUC), calibration, and clinical utility (decision-curve analysis, DCA). Interpretability combined a nomogram with Shapley additive explanations (SHAP); a bilingual web calculator was also implemented.

**Results:**

Of 799 PPMI participants, 169 (21.2%) met the MoCA-based PD-MCI definition. Seven routinely collected predictors were retained (sex, age, education, age at disease onset, MDS-UPDRS Part III, GDS, UPSIT). LR showed the most balanced performance: AUC 0.789 (training), 0.778 (internal), and 0.772 (external). At a fixed threshold of 0.50 in the external cohort, LR’s sensitivity was 89.7%, specificity 43.9%, and F1-score 66.7%. Calibration and DCA favored LR. SHAP indicated education and motor severity as dominant contributors, followed by sex and age at onset; depressive burden (GDS) and hyposmia (UPSIT) increased risk, whereas chronological age had a smaller marginal effect.

**Conclusions:**

We developed and externally validated a probability-based, clinic-ready risk-stratification tool for PD-MCI using routinely available variables and MoCA-based MDS Level I labels. Implemented as a nomogram and bilingual calculator, it supports sensitivity-oriented triage—especially among MoCA-normal patients—by prioritizing timely MDS Level II evaluation and closer follow-up. The tool complements, rather than replaces, formal diagnostic assessment and does not predict long-term conversion.

**Clinical trial number:**

Not applicable. The PPMI study is registered with ClinicalTrials.gov (NCT01141023) and the registration date is June 8, 2010.

**Supplementary Information:**

The online version contains supplementary material available at 10.1186/s12911-025-03215-0.

## Introduction

Parkinson’s disease (PD), the second most prevalent neurodegenerative disorder following Alzheimer’s disease (AD), is clinically characterized by progressive motor dysfunction. Among individuals aged 65 years and older, the global prevalence exceeds 1%, with projections indicating that this burden will approximately double by 2030 [1]. In addition to its hallmark motor symptoms, PD encompasses a broad range of non-motor manifestations; cognitive impairment stands out as one of the most common and clinically significant issues, exerting considerable effects on quality of life, survival rates, and healthcare utilization [2–4].

Mild cognitive impairment (MCI) is characterized as a transitional phase between cognition that aligns with age and education expectations and the onset of dementia. This condition is marked by an objective decline in cognitive abilities while basic daily functioning remains intact [5–7]. In the context of Parkinson’s disease (PD), this phenomenon is referred to as PD-MCI. Current estimates suggest that approximately 19% to 38% (mean ∼ 27%) of non-demented PD patients meet the criteria for PD-MCI [8, 9]. Longitudinal studies indicate a significantly higher risk of conversion to Parkinson’s disease dementia (PDD) compared to cognitively normal PD patients (PD-NC) [8, 10, 11]. The cognitive decline associated with PD-MCI not only diminishes patients’ quality of life but also imposes substantial socioeconomic burdens on caregivers and healthcare systems, while potentially contributing to increased mortality [12, 13]. These findings underscore the critical importance of early identification of PD patients at elevated risk for cognitive decline.

Current approaches to identifying and characterizing PD-MCI fall into two broad categories. First, cognitive assessment is typically performed with the Montreal Cognitive Assessment (MoCA) under the Movement Disorder Society (MDS) Level I framework, whereas definitive characterization relies on comprehensive Level II neuropsychological evaluation [6, 14]. However, cognitive instruments primarily detect established impairment; MoCA, in particular, dichotomizes current status and does not quantify risk among patients who do not reach the PD-MCI cutoff. Second, biomarker-oriented approaches (e.g., imaging measures, cerebrospinal fluid analyses, olfactory tests) are scientifically informative but often difficult to deploy at scale for everyday triage across diverse clinics, and many studies evaluate single markers outside a comprehensive decision framework [15]. Taken together, these considerations point to a practical need: an interpretable, clinic-ready way to estimate PD-MCI risk among patients who screen MoCA-normal, so that the intensity of follow-up and the timing of diagnostic work-ups can be prioritized appropriately.

Meeting this need necessitates both a robust data substrate and methodologies capable of synthesizing it. Large, harmonized PD cohorts—such as the Parkinson’s Progression Markers Initiative (PPMI) database—provide a diverse case mix, standardized multidomain phenotyping, and sample sizes that are conducive to model development and out-of-sample testing [16, 17]. Concurrently, high-volume and high-dimensional clinical data present challenges for traditional analyses due to nonlinearities, interactions, and multicollinearity. Machine learning models are particularly well-equipped to integrate such multidimensional signals and yield individualized probabilities rather than binary classifications; this capability supports nuanced decision-making in real-world contexts [18–20]. Insights from clinical prediction research further underscore the importance of balancing model complexity with interpretability and transportability. Transparent models that undergo rigorous internal/external validation, calibration assessment, and decision-curve evaluation are more likely to generalize effectively and gain acceptance at the point of care [21, 22].

To the best of our knowledge, there is a limited availability of large-scale, externally validated machine learning models specifically designed for the risk stratification of PD-MCI. Therefore, we used the large, well-phenotyped PPMI cohort to develop and externally validate a probability-based risk-stratification model. By leveraging routinely obtainable clinical variables, the model aims to identify patients at elevated risk of PD-MCI—particularly those with normal MoCA scores at the current evaluation—to guide timely MDS Level II diagnosis and subsequent management strategies.

## Methods

### Study population and design

We conducted a cross-sectional study utilizing data from the PPMI database (www.ppmi-info.org). The training and internal validation cohorts were derived from the PPMI_Curated_Data_Cut_Public_20230612_rev. PPMI is a multicenter observational study that recruits patients with PD through specialized movement disorder clinics across North America and Europe, with the aim of identifying biomarkers associated with PD heterogeneity and progression. The study protocol received approval from the institutional review board at each participating site (ClinicalTrials. gov Identifier: NCT01141023, Registration Date: June 8, 2010).

For external validation, we enrolled 70 consecutive PD patients from The Affiliated Hospital of Guilin Medical University between February 2024 and March 2025. This study was approved by the hospital’s Ethics Committee (Approval No. 2025IITLL-27), and all participants provided written informed consent. The study adhered to the principles outlined in the Declaration of Helsinki.

### Inclusion and exclusion criteria

Participants were screened according to the following criteria:

Inclusion criteria:

1. Diagnosis of PD according to the MDS Clinical Diagnostic Criteria [23];
2. Availability of complete MoCA results.

Exclusion criteria:

1. MoCA score < 21, suggestive of probable dementia rather than MCI;
2. Missing cognitive assessment data;
3. Implausible or clinically inconsistent MoCA scores.

### Cognitive classification

Cognitive status was operationally defined in accordance with the Level I diagnostic criteria for PD-MCI proposed by the MDS Task Force [6]. This operational definition translates the clinical concept of MCI into explicit and reproducible criteria, ensuring consistent application across various centers and studies (MoCA scores were education-adjusted (+ 1 point for ≤ 12 years of education) before applying PD-MCI thresholds):

1. PD-NC: MoCA score of 26–30;
2. PD-MCI: MoCA score of 21–25.

This MoCA-based classification has been validated in recent large-scale cohorts of individuals with PD, demonstrating its sensitivity and specificity for detecting early cognitive changes when comprehensive neuropsychological testing is not feasible [24, 25].

### Data processing and modeling

**Fig. 1:**
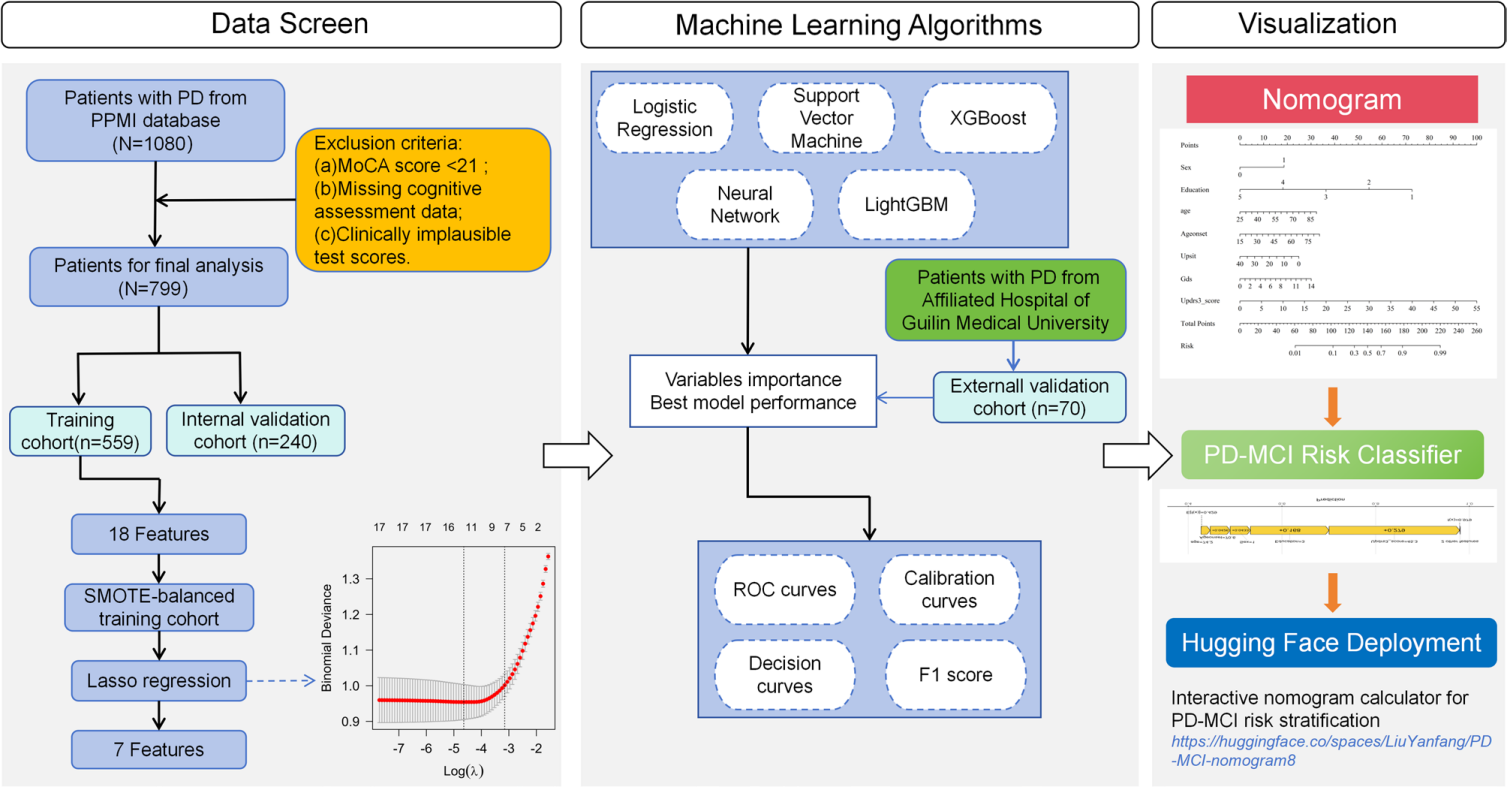
The overall flowchart of the study

The workflow is illustrated in Fig. 1. All analyses were performed in R version 4.4.3.

#### Variable screening and preprocessing

Prior to model training, variable selection was performed to reduce noise and mitigate overfitting by excluding irrelevant or redundant variables [26]. Variables were excluded if they met any of the following criteria:

1. More than 20% missing data;
2. No established association with MCI in PD, based on prior research or clinical expertise;
3. Not derived from validated clinical assessments, imaging studies, or laboratory tests.

For reproducibility, clinical variable names are followed by the dataset variable in parentheses; units are given where applicable. The final variable set included:

1. Demographic/clinical: age (age; years), sex (sex), education level (Education; 5-level ordinal: 1 = primary school, 2 = middle school, 3 = high school, 4 = university, 5 = graduate), body mass index (BMI; kg/m²), family history of PD (fampb_bin; 1 = yes, 0 = no), age at disease onset (ageonset; years), disease duration (Duration; years).
2. Motor symptoms: Hoehn & Yahr stage (H&Y; variable NHY), MDS-UPDRS Part III motor score (MDS-UPDRS III; variable Updrs3_Score), tremor-dominant vs. postural instability/gait disorder phenotype (TD/PIGD; variable td_pigd).
3. Non-motor symptoms: Geriatric Depression Scale (GDS; variable Gds), State–Trait Anxiety Inventory total (STAI-total; variable Stai) and subscales (STAI-State, STAI-Trait; variable Stai_State, Stai_Trait), Scales for Outcomes in Parkinson’s Disease-Autonomic total score (SCOPA-AUT; variable Scopa), Epworth Sleepiness Scale (ESS; variable Ess), REM Sleep Behavior Disorder Questionnaire (REM; variable Rem), University of Pennsylvania Smell Identification Test (UPSIT; variable Upsit).

Missing data were imputed using multiple imputation by chained equations (MICE) [27].

#### Handling class imbalance

Given the lower prevalence of PD-MCI [8, 9], the Synthetic Minority Over-sampling Technique (SMOTE) was exclusively utilized for the training cohort to address class imbalance, whereas the internal and external validation cohorts were left unchanged to maintain authentic class distributions and avoid resampling bias [28].

#### Regularized feature selection

Feature selection was performed on the SMOTE-adjusted training cohort utilizing Least Absolute Shrinkage and Selection Operator (LASSO) regression with 10-fold cross-validation [26], retaining variables with non-zero coefficients at the optimal λ determined by the 1-SE criterion.

#### Model development

Five supervised machine learning models were selected to represent both interpretable linear and advanced nonlinear approaches:

1. Logistic Regression (LR): widely used as a baseline in clinical prediction for its high interpretability [29];
2. Support Vector Machine (SVM): effective in high-dimensional feature spaces and robust against outliers [30];
3. Extreme Gradient Boosting (XGBoost): a gradient-boosting framework optimized for speed and predictive accuracy, well-suited for structured biomedical data [31];
4. Neural Network (NN): capable of modeling complex nonlinear interactions among predictors [32];
5. Light Gradient Boosting Machine (LightGBM): a computationally efficient gradient-boosting method suitable for large or high-dimensional datasets [33].

All models were trained on the SMOTE-adjusted training cohort. Hyperparameters were optimized using grid search with 10-fold cross-validation, maximizing area under the receiver operating characteristic curve (ROC-AUC) in the training cohort [34].

#### Model evaluation

No validation data were used for model fitting or feature selection, in accordance with Transparent Reporting of a Multivariable Prediction Model for Individual Prognosis or Diagnosis (TRIPOD) guidelines [21]. All performance evaluations were conducted on the original (non-resampled) datasets: the original training cohort, the internal validation cohort, and the external validation cohort. We evaluated model performance using the following metrics:

1. Discrimination: AUC [35] with 95% confidence intervals was estimated using the DeLong method [36]. All AUCs were computed from full ROC curves on non-resampled data. For the training cohort, ROC/AUC were computed on the original (non-resampled) cohort (SMOTE was used only during model fitting).
2. Calibration: Bootstrap bias-corrected calibration plots (1,000 resamples) and Hosmer-Lemeshow tests conducted in the internal and external validation cohorts [37].
3. Clinical utility: Decision curve analysis (DCA) [36] in internal and external validation cohorts, with threshold range 0–1 (step = 0.01).
4. Threshold-based assessment: Sensitivity, specificity, accuracy, and F1 score were computed at: (a) a fixed cut-off of 0.50, in all three datasets evaluated on native (non-resampled) distributions; and (b) the Youden-optimal cut-off [38], determined by cross-validated ROC analysis in the original training cohort.

### Model interpretation

To ensure a transparent and clinically relevant interpretation of the final risk-stratification model, we employed two complementary approaches alongside a pragmatic banding scheme.

1. Nomogram: Using the rms package in R, LR coefficients were transformed into a points system wherein each predictor contributes points proportional to its coefficient. The total points are subsequently mapped to the model-based probability of the MoCA-based MDS Level I PD-MCI label at the current evaluation [39].
2. SHAP: Using the iml package in R, SHAP values were computed to quantify each predictor’s marginal contribution to an individual’s estimated risk. This facilitates both global importance rankings and case-level explanations through summary, dependence, and force plots [40].
3. Probability bands: For descriptive clinical interpretation, predicted probabilities were categorized as low (< 0.30), intermediate (0.30–<0.60), and high (≥ 0.60) according to published risk communication guidance [41]; these bands were descriptive only and not used for model fitting.

The final nomogram was also deployed on the Hugging Face Spaces platform as an interactive bilingual (English/Chinese) web calculator, enabling individualized PD-MCI risk estimation and model-based recommendations.

### Statistical analysis

Continuous variables were summarized as mean ± SD or median (IQR) and compared using Student’s t-test or Wilcoxon rank-sum test. Categorical variables were compared using chi-square or Fisher’s exact test. Two-sided p-values < 0.05 were considered statistically significant. ROC, calibration, and DCA analyses were performed with the pROC, rms, and rmda packages.

## Results

### Participant characteristics

The study analyzed 799 PD cases from the PPMI database as our development cohort, with detailed clinical characteristics presented in Table 1. This cohort had a mean age of 62.90 years and included 447 males (55.9%), demonstrating a PD-MCI prevalence of 21.15%. Participants were randomly divided into training (*n* = 559) and internal validation (*n* = 240) cohorts at a 7:3 ratio. The training cohort exhibited a PD-MCI prevalence of 20.04% (112 patients), while the internal validation cohort showed a prevalence of 23.75% (57 patients).

For external validation, we enrolled 70 PD patients from The Affiliated Hospital of Guilin Medical University (February 2024–March 2025). This cohort exhibited a higher PD-MCI prevalence (41.43%, 29 patients) compared to the development cohort (21.15%), with 58.57% of patients remaining cognitively normal. Additionally, the external cohort was younger, with a mean age of 58.2 years and earlier disease onset, which may explain the observed differences in sensitivity and specificity relative to the training cohort. Detailed demographic and clinical comparisons are shown in Table 1.

**Table 1:** Baseline characteristics of study participants

Variables	PPMI cohort (*n* = 799)	Training cohort (*n* = 559)	Internal validation cohort (*n* = 240)	External validation cohort (*n* = 70)	*P*
Age, years	62.90 ± 9.98	63.25 ± 9.76	62.07 ± 10.44	58.43 ± 7.85	**< 0.001** ^*^
BMI, kg/m²	26.53 ± 4.88	26.68 ± 4.96	26.20 ± 4.68		0.204^#^
Age at onset, years	60.95 (53.44–67.71)	61.28 (53.66–67.98)	59.93 (52.56–66.69)	52.50 (46.00–60.75)	**< 0.001** ^*^
Disease duration, years	7.17 (3.65–19.65)	7.03 (3.63–19.32)	7.47 (3.83–20.88)		0.261^#^
UPSIT	22.00 (15.00–27.00)	22.00 (15.00–27.00)	22.00 (15.00–28.00)	20.00 (14.00–25.75)	0.245^*^
ESS	5.00 (3.00–8.00)	5.00 (3.00–8.00)	6.00 (3.00–8.00)		0.298^#^
REM	3.00 (2.00–6.00)	3.00 (2.00–5.00)	3.00 (2.00–6.00)		0.061^#^
GDS	2.00 (1.00–3.00)	2.00 (1.00–3.00)	2.00 (1.00–4.00)	1.00 (1.00–3.00)	0.272^*^
STAI total	61.00 (50.00–77.00)	61.00 (49.00–76.00)	62.00 (51.00–79.00)		0.357^#^
STAI-State	30.00 (24.00–40.00)	30.00 (24.00–39.00)	30.50 (24.00–40.00)		0.717^#^
STAI-Trait	31.00 (25.0–38.00)	30.00 (24.00–37.00)	31.00 (25.00–39.00)		0.196^#^
SCOPA-AUT	9.00 (6.00–14.00)	9.00 (6.00–14.00)	10.00 (6.00–14.00)		0.371^#^
MDS-UPDRS III	21.00 (15.00–28.00)	20.00 (15.00–28.00)	22.00 (15.00–29.25)	22.00 (16.00–28.00)	0.409^*^
Sex					0.163^*^
Female	352 (44.06%)	250 (44.72%)	102 (42.50%)	23 (32.86%)	
Male	447 (55.94%)	309 (55.28%)	138 (57.50%)	47 (67.14%)	
Family history of PD					**0.031** ^#^
Yes	269 (33.67%)	175 (31.31%)	94 (39.17%)		
No	530 (66.33%)	384 (68.69%)	146 (60.83%)		
TD/PIGD					**0.024** ^#^
TD	544 (68.09%)	367 (65.65%)	177 (73.75%)		
PIGD	255 (31.91%)	192 (34.35%)	63 (26.25%)		
H&Y stage					0.110^#^
Stage 1	274 (34.29%)	189 (33.81%)	85 (35.42%)		
Stage 2	455 (56.95%)	328 (58.68%)	127 (52.92%)		
Stage 3	70 (8.76%)	42 (7.51%)	28 (11.67%)		
Education				-	**< 0.001** ^*^
1	3 (0.38%)	1 (0.18%)	2 (0.83%)	12 (17.14%)	
2	5 (0.63%)	2 (0.36%)	3 (1.25%)	20 (28.57%)	
3	76 (9.51%)	49 (8.77%)	27 (11.25%)	29 (41.43%)	
4	365 (45.68%)	257 (45.97%)	108 (45.00%)	9 (12.86%)	
5	350 (43.80%)	250 (44.72%)	100 (41.67%)	0 (0.00%)	
Cognitive status					**< 0.001** ^*^
PD-NC	630 (78.85%)	447 (79.96%)	183 (76.25%)	41 (58.57%)	
PD-MCI	169 (21.15%)	112 (20.04%)	57 (23.75%)	29 (41.43%)	

Continuous: variables are reported as mean ± SD or median (Q1–Q3), as appropriate; Categorical variables are n (%). *P* values: *Three-group comparisons (training vs. internal vs. external) were conducted for the seven candidate predictors; ^#^Two-group comparisons (training vs. internal) were performed for the other predictors. Values reflect original (unresampled) data; SMOTE was applied only within the training cohort during model development

Abbreviations: BMI, body mass index; UPSIT, University of Pennsylvania Smell Identification Test; ESS, Epworth Sleepiness Scale; REM, REM Sleep Behavior Disorder Questionnaire; GDS, Geriatric Depression Scale; STAI, State–Trait Anxiety Inventory (total, State, Trait); SCOPA-AUT, Scales for Outcomes in Parkinson’s Disease–Autonomic; MDS–UPDRS III, Movement Disorder Society–Unified Parkinson’s Disease Rating Scale Part III; H&Y, Hoehn & Yahr stage; TD/PIGD, tremor-dominant vs. postural instability/gait disorder phenotype. Cognitive status, MoCA-defined cognitive status (PD-NC vs. PD-MCI)

Education coding: 1 = Primary, 2 = Middle, 3 = High school, 4 = University, 5 = Graduate

### Feature selection and candidate predictors

**Fig. 2:**
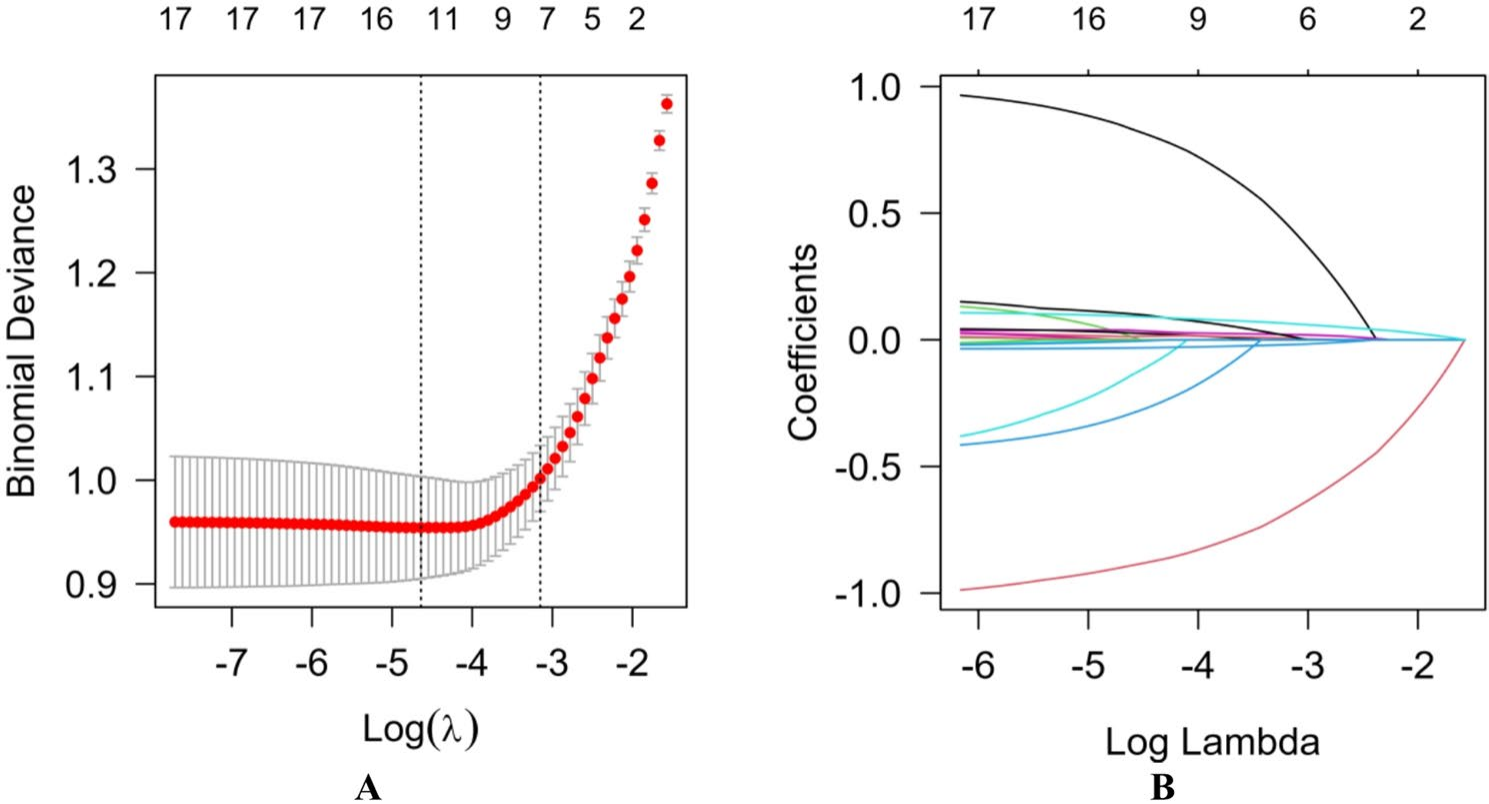
LASSO feature selection in the SMOTE-adjusted training cohort. (**A**) 10-fold cross-validated binomial deviance plotted against log(λ); vertical dashed lines mark λ_min and λ_1SE, with the 1-SE criterion used for feature selection. (**B**) Coefficient paths across log(λ); at the 1-SE criterion, seven predictors retain non-zero coefficients: sex, age, education level, age at disease onset, UPSIT, GDS, and MDS-UPDRS III. Abbreviations: UPSIT, University of Pennsylvania Smell Identification Test; GDS, Geriatric Depression Scale; MDS-UPDRS III, Movement Disorder Society–Unified Parkinson’s Disease Rating Scale part III

Using 10-fold cross-validated LASSO on the SMOTE-adjusted training cohort, we identified seven predictors at the 1-SE criterion (Fig. 2A–B): sex, age, education level, age at disease onset, UPSIT, GDS, and MDS-UPDRS III.

### Development and validation of machine-learning models

**Fig. 3:**
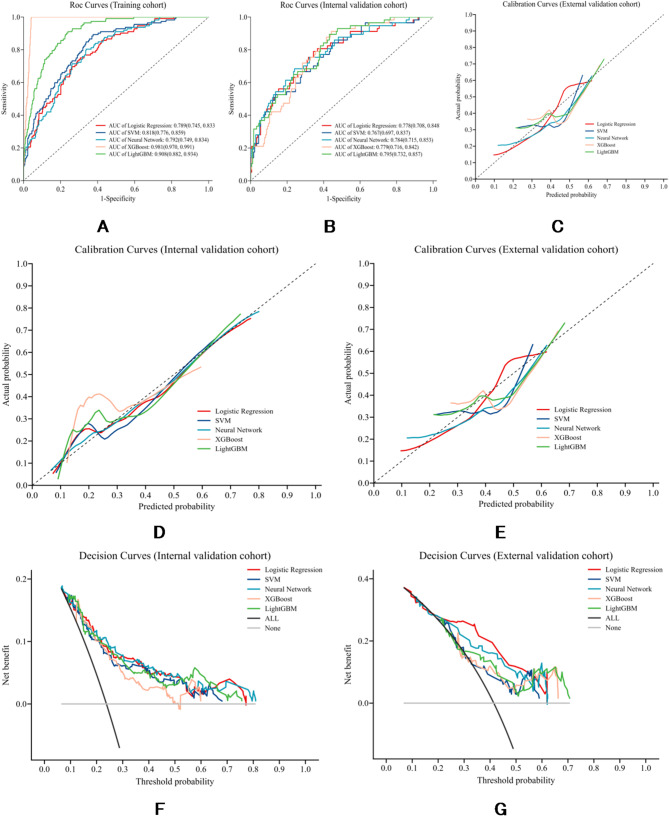
The performance and comparison of five models in the training, internal validation, and external validation cohorts. (**A**–**C**) Receiver operating characteristic (ROC) curves in the training (**A**), internal validation (**B**), and external validation (**C**) cohorts. (**D**-**E**) Calibration curves in the internal (**D**) and external (**E**) validation cohorts. (**F**-**G**) Decision curve analysis (DCA) in the internal (**F**) and external (**G**) validation cohorts

Using the SMOTE-adjusted training cohort, we developed five models (LR, SVM, XGBoost, NN, LightGBM) and evaluated them on the original (non-resampled) training, internal, and external cohorts. ROC curves for all three cohorts are shown in Fig. 3A–C; calibration and decision-curve analyses (DCA) were prespecified for validation cohorts (Fig. 3D–G). Threshold-based metrics at a fixed cutoff of 0.50 are summarized in Table 2.

Across models, LR showed the most balanced and generalizable performance. In the external cohort, LR achieved the highest AUC (0.772, 95% CI 0.657–0.887) and the highest F1 score (66.7%), with high sensitivity (89.7%) but lower specificity (43.9%). Performance was stable from development to validation (training 0.789 [95% CI 0.745–0.833]; internal 0.778 [95% CI 0.705–0.848]) without signs of overfitting. By design, SMOTE was applied only during model fitting while preserving native class distributions in validation, yielding a sensitivity-oriented operating profile appropriate for risk stratification.

In contrast, tree-boosting models exhibited optimism that did not transfer externally (e.g., XGBoost: training AUC 0.981, external AUC 0.685; LightGBM: training AUC 0.908, external AUC 0.701), and SVM/NN underperformed relative to LR. LR also demonstrated the closest alignment to observed risks on calibration and the greatest net benefit across a wide threshold range on DCA (Fig. 3D–G). Based on convergent evidence across discrimination, calibration, and clinical utility, LR was selected as the final model for interpretation and deployment. Additional model performance metrics across five algorithms at Youden-optimal cutoffs are provided in Supplementary Table S1, and detailed LR coefficients/odds ratios in Supplementary Table S2.

### Operating threshold for the logistic regression model

**Fig. 4:**
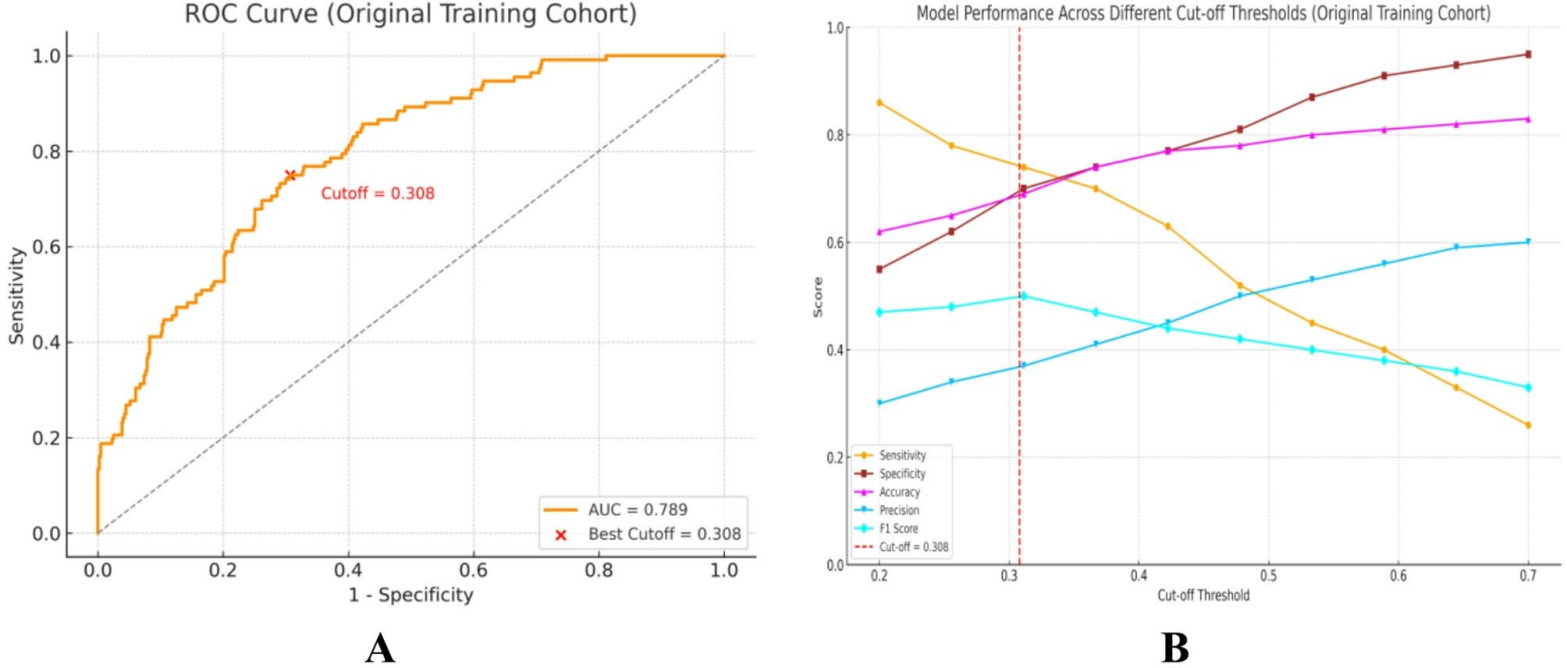
Final logistic regression model operating characteristics in the original training cohort. (**A**) ROC curve. (**B**) Threshold-wise sensitivity, specificity, F1, and Youden index; cross-validated Youden-optimal threshold = 0.308

Utilizing the original (non-resampled) training cohort, we systematically examined the entire range of probability thresholds for the final LR model. The ROC curve and threshold performance profiles are illustrated in Fig. 4A–B. As the threshold was reduced from 0.50 to approximately 0.30, sensitivity increased while specificity decreased; both F1 score and the Youden index reached their peak around thresholds of 0.35 to 0.40. Cross-validated analysis determined a Youden-optimal threshold of 0.308 within the training cohort. This analysis complements the unified comparison at a threshold of 0.50 presented in Table 2 by demonstrating how varying operating points influence the sensitivity-specificity balance in clinical triage.

**Table 2:** Model performance across cohorts: ROC AUCs (threshold-free) and threshold-based metrics at probability cut-off 0.50

Cohort	Index	Logistic regression	XGBoost	LightGBM	SVM	Neural network
Training	Accuracy	78.2%	94.8%	85.3%	78.2%	77.6%
	AUC	0.789	0.981	0.908	0.818	0.792
	Sensitivity	47.3%	100.0%	67.9%	57.1%	42.0%
	Specificity	85.9%	93.5%	89.7%	83.4%	86.6%
	Precision	45.7%	79.4%	62.3%	46.4%	43.9%
	F1	46.5%	88.5%	65.0%	51.2%	42.9%
Internal validation	Accuracy	77.5%	72.1%	75.8%	73.8%	78.7%
	AUC	0.778	0.779	0.795	0.767	0.784
	Sensitivity	56.1%	47.4%	52.6%	54.4%	54.4%
	Specificity	84.2%	79.8%	83.1%	79.8%	86.3%
	Precision	52.5%	42.2%	49.2%	45.6%	55.4%
	F1	54.2%	44.6%	50.8%	49.6%	54.9%
External validation	Accuracy	62.9%	61.4%	64.3%	57.1%	61.4%
	AUC	0.772	0.685	0.701	0.675	0.749
	Sensitivity	89.7%	48.3%	55.2%	72.4%	86.2%
	Specificity	43.9%	70.7%	70.7%	46.3%	43.9%
	Precision	53.1%	53.8%	57.1%	48.8%	52.1%
	F1	66.7%	50.9%	56.1%	58.3%	64.9%

Note: AUCs were computed from full ROC curves on non-resampled data. Sensitivity, specificity, accuracy, and F1 were calculated at a fixed probability cut-off of 0.50

### Nomogram and point-of-care calculator

**Fig. 5:**
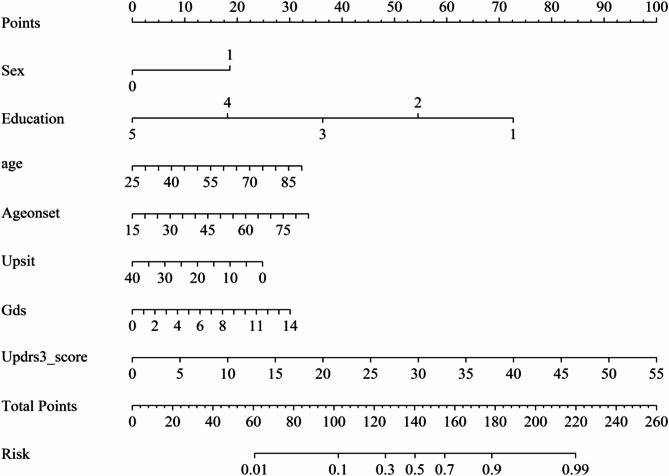
Nomogram for the logistic regression model integrating seven predictors; total points map to the model-estimated probability of MoCA-defined PD-MCI at the current evaluation. Abbreviations: Education, level of formal education; Ageonset, age at disease onset; UPSIT, University of Pennsylvania Smell Identification Test; GDS, Geriatric Depression Scale; Updrs3_score, Movement Disorder Society–Unified Parkinson’s Disease Rating Scale Part III. Variable Coding: Sex: 0 = Female, 1 = Male; Education: 1 = Primary, 2 = Middle, 3 = High school, 4 = University, 5 = Graduate

The final LR model is presented as a nomogram that integrates seven predictors: sex, age, education level, age at onset, UPSIT, GDS, and MDS-UPDRS III. Each predictor contributes points in proportion to its respective regression coefficient; the total score maps to the model-estimated probability of meeting the MoCA-based PD-MCI definition (Fig. 5). An illustrative nomogram-based calculation for a high-risk patient is presented in Supplementary Fig. S1.

To enhance point-of-care usability without necessitating manual scoring, we have also developed a functionally equivalent bilingual web calculator available on Hugging Face (https://huggingface.co/spaces/LiuYanfang/PD-MCI-nomogram8). The tool accepts the same seven inputs and returns both the model-estimated probability along with prespecified bands (low < 0.30, intermediate 0.30–<0.60, high ≥ 0.60). To assist with clinical triage, the interface additionally offers band-contingent suggestions (e.g., consideration of comprehensive cognitive testing, review of anticholinergic/sedative medications, tailored follow-up intervals, and lifestyle or rehabilitation advice). It is crucial to emphasize that this tool is intended to support risk stratification and clinical triage rather than replace MDS Level II diagnosis. Screenshots for different risk levels are shown in Supplementary Fig. S2.

**Fig. 6:**
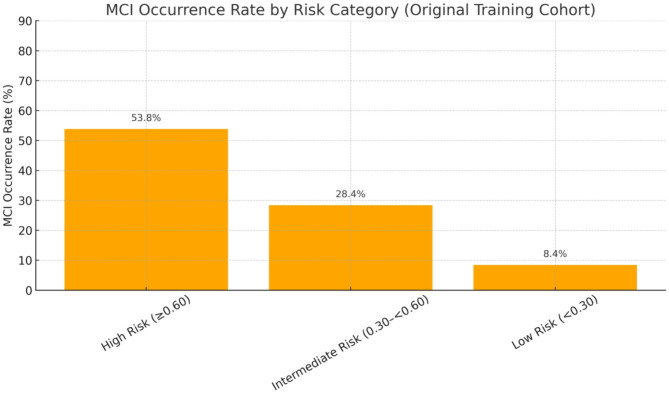
Observed PD-MCI proportions by prespecified probability bands in the original training cohort: low (< 0.30) = 8.4%, intermediate (0.30–<0.60) = 28.4%, high (≥ 0.60) = 53.8%

### Risk-band yields in the original training cohort

Applying the prespecified probability bands to the original (non-resampled) training cohort, we observed a monotonic increase in the proportions of MoCA-defined PD-MCI across risk bands: 8.4% in the low-risk group (< 0.30), 28.4% in the intermediate group (0.30–<0.60), and 53.8% in the high-risk group (≥ 0.60) (Fig. 6). These findings suggest that the risk bands effectively stratify patients into groups with significantly different probabilities of PD-MCI, thereby supporting their intended application for risk stratification and prioritization of follow-up intensity.

### SHAP-based interpretation of the logistic regression model

**Fig. 7:**
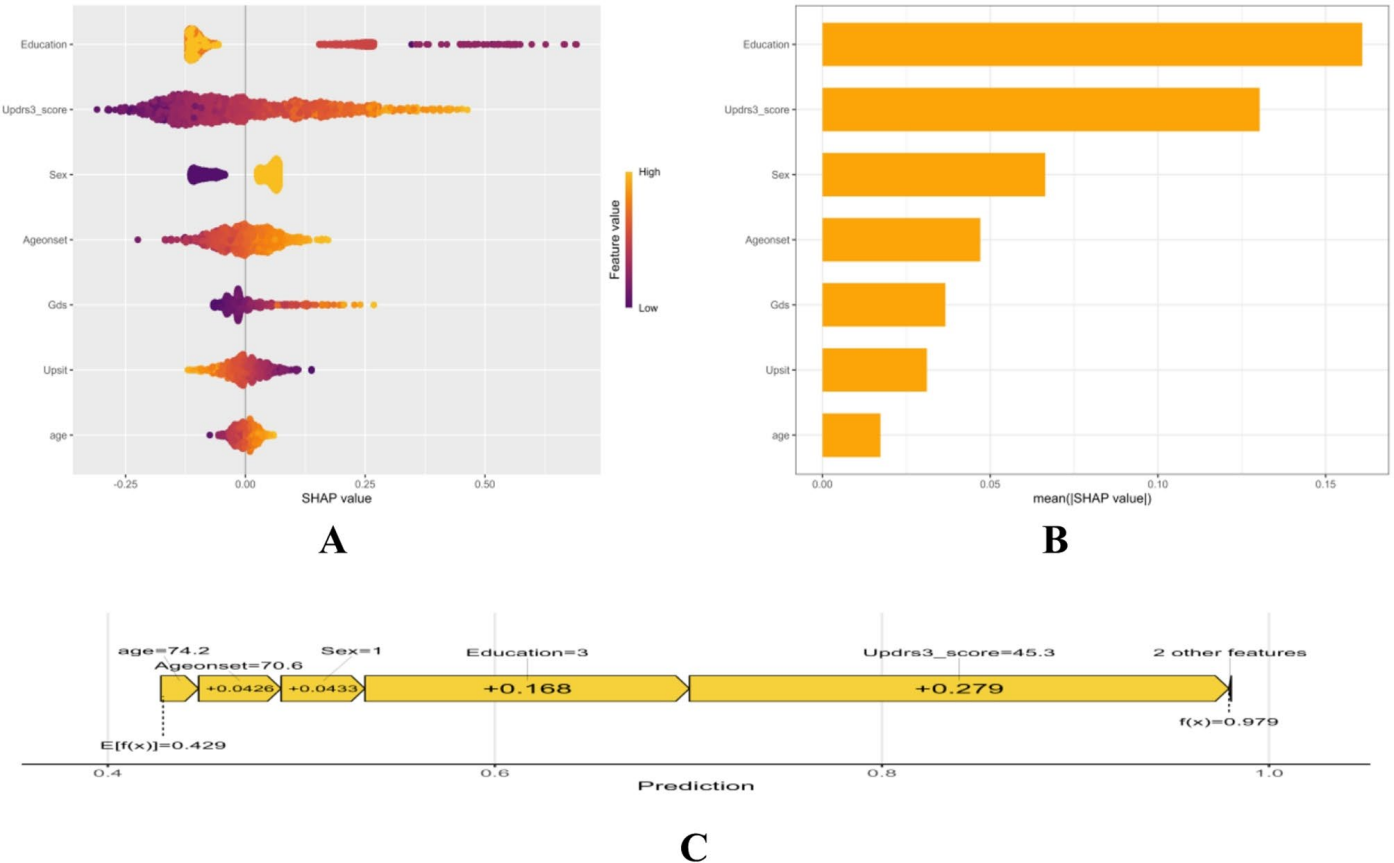
SHAP-based interpretation of the logistic regression model. (**A**) SHAP summary plot. Each point represents a sample. The x-axis shows the SHAP value, indicating the impact of each feature on model output. Color reflects the original feature value, mapped continuously from low to high based on the color scale. (**B**) Feature importance ranked by the mean absolute SHAP value. (**C**) SHAP force plot illustrating the feature contributions to the predicted risk for a representative patient

The SHAP summaries for the LR model highlights the following global importance (mean |SHAP|): education, MDS-UPDRS III, sex, age at onset, GDS, UPSIT, and age (Fig. 7B). The SHAP value “swarm” plot (Fig. 7A) shows that lower education levels, higher MDS-UPDRS III scores, male sex, later ages at onset, increased GDS values, and lower UPSIT scores tend to elevate the risk estimate; while age has a minor effect. A case-level force plot (Fig. 7C) illustrates attribution for a representative high-risk individual. In this instance, substantial positive contributions from MDS-UPDRS III (+ 0.279) and lower education (+ 0.168), along with smaller positive influences from sex and age at onset, shift the prediction from the cohort baseline (E[f(x)] = 0.429) to 0.979. These insights from SHAP enhance model transparency and provide clinically meaningful interpretations for individualized risk estimation.

## Discussion

Cognitive impairment is a common and clinically significant non-motor manifestation of PD, which is associated with poorer quality of life, increased mortality rates, and heightened healthcare utilization [2–4]. Meta-analytic estimates indicate that approximately one quarter of non-demented patients meet criteria for PD-MCI [8, 9], broadly consistent with the proportions observed in our development and internal validation cohorts. Given that PD-MCI is a strong precursor to PDD, demonstrating a significantly higher conversion risk compared to cognitively normal PD, the early identification of patients at increased risk for PD-MCI has substantial clinical implications for care planning [42].

While the MoCA (MDS Level I) is widely employed to screen for current cognitive impairment, it does not effectively quantify risk among patients who do not meet the cutoff criteria for PD-MCI. To address this gap, we developed and externally validated a probability-based risk-stratification tool that utilizes routinely obtainable clinical variables to identify MoCA-normal patients at an increased risk—thereby assisting clinicians in determining which individuals should undergo timely MDS Level II diagnosis, closer follow-up, and preventive or mitigating measures (e.g., medication review concerning cognitive side effects, referral to neuropsychology services, targeted rehabilitation). Although the model was trained and evaluated on the complete cohorts (PD-NC + PD-MCI), its greatest incremental utility in clinical practice is observed among patients who are MoCA-normal at the time of assessment. Importantly, to handle class imbalance without distorting prevalence, SMOTE was applied only during model fitting; both validation cohorts retained native class distributions to yield honest out-of-sample estimates. In this subgroup, a higher probability estimated by the model does not serve as a substitute for a formal diagnosis; rather, it assists clinicians in prioritizing which patients should undergo timely MDS Level II diagnosis and receive closer follow-up. This tool is designed as an adjunct for triage and shared decision-making rather than serving as a substitute for MDS Level II diagnosis or acting as a predictor of long-term conversion.

We compared five supervised models in a large, well-phenotyped development cohort (PPMI) and evaluated them in both an internal cohort and an independent external cohort. LR demonstrated the most balanced and generalizable profile: discrimination was only modestly attenuated from development to validation, calibration in the validation cohorts closely tracked the ideal line, and DCA indicated a higher net benefit across a broad range of thresholds compared to alternative models (Table 2; Fig. 3D–G). For operational triage—where minimizing missed higher-risk cases is prioritized—this sensitivity-oriented operating profile is appropriate. In contrast, tree-boosting models (XGBoost, LightGBM) exhibited stronger apparent performance during development but transferred less effectively to the external cohort, which aligns with patterns of optimism/overfitting observed in structured clinical data; similarly, SVM and NN did not match LR on external discrimination or F1 scores at the common cutoff of 0.50 (Table 2). Based on these considerations, we selected LR as the final model for downstream interpretation and clinical translation, given its transportability, calibration, net-benefit profile, parsimony, and ease of implementation.

Placed alongside the broader literature on PD cognition and PD-MCI, most studies have emphasized three distinct aims: (i) mechanistic and pathophysiological syntheses that link Lewy co-pathologies (β-amyloid and tau), progressive cholinergic denervation, and frontostriatal network disruption with age-related vulnerability [43, 44]; (ii) discovery of single biomarkers—such as imaging networks and cerebrospinal fluid/olfactory measures—that are scientifically informative but less readily deployable at scale for routine triage [15, 45, 46]; and (iii) diagnostic confirmation frameworks that standardize MDS Level I/II criteria while prioritizing formal neuropsychological assessment rather than probability-based risk stratification among MoCA-normal patients [6, 14]. Our study diverges from these approaches in four clinically relevant ways. First, it addresses the triage problem by training models on the complete labeled spectrum of MoCA Level I PD-NC and PD-MCI to mitigate spectrum bias. This approach enables us to learn a calibrated probability function primarily intended for use in MoCA-normal patients—where such calibration is particularly valuable for prioritizing timely MDS Level II diagnosis and closer follow-up. Second, to enhance deployability beyond specialized centers, we intentionally restricted inputs to routinely collected variables—including age, age at onset, education level, sex, MDS-UPDRS III, GDS, and UPSIT—instead of relying on advanced imaging or cerebrospinal fluid markers [15, 44–46]. Third, we prespecified both internal validation procedures as well as independent external validation metrics; we reported discrimination performance along with calibration assessments and decision-curve net benefits in accordance with contemporary guidelines for clinical prediction models [21–37]. Finally, we underscore interpretability and practical application: following a head-to-head comparison of five supervised learning models, LR was selected for downstream implementation due to its superior balanced external performance; its transparency and straightforward implementation further support clinical translation via a nomogram, SHAP explanations, and prespecified probability bands, together with a bilingual web calculator aligned to the locked coefficients.

Beyond aggregate metrics, interpretability was an a priori requirement. The nomogram (Fig. 5) renders the final LR into a points-based graphic, allowing clinicians to visualize how each predictor contributes and to obtain a probability estimate without computation. SHAP summaries add a model-agnostic explanation layer (Fig. 7): globally, education and motor severity (MDS-UPDRS III) contributed most, followed by sex and age at onset, with depressive burden (GDS) and hyposmia (UPSIT) also increasing the estimate; chronological age showed a smaller marginal contribution in this LR specification. These directions are biologically and clinically coherent. Older age and later onset likely reflect cumulative Lewy co-pathologies (e.g., β-amyloid, tau) and progressive cholinergic denervation, which reduce neural/cognitive reserve and increase vulnerability to inefficiency [47, 48]. Greater motor burden (higher MDS-UPDRS III) aligns with frontostriatal and cholinergic network dysfunction and often tracks broader extranigral pathology relevant to cognition [47, 49]. Depressive symptoms (higher GDS) co-travel with poorer attention, working memory, and learning in PD—plausibly via limbic–frontostriatal dysregulation and monoaminergic/cholinergic changes [50–52]. Hyposmia (lower UPSIT) is a robust marker of extranigral Lewy pathology in olfactory/limbic cortices and predicts higher risk of cognitive involvement and faster decline in longitudinal cohorts [53–55]. Lower educational attainment is consistent with the cognitive-reserve framework, whereby reduced reserve diminishes the capacity to compensate for neuropathology [56, 57]. Male sex has been associated with higher cognitive risk in several datasets, potentially reflecting lower estrogenic neuroprotection and sex-linked vascular–metabolic profiles [50, 58]. At the individual level, the SHAP panel (Fig. 7C) makes the estimate auditable by showing how “low UPSIT + high motor/depressive burden” push risk upward while higher education partially offsets that effect—supporting shared decision-making.

To support deployment, we complemented discrimination with threshold-based evaluation. In our dataset, operating points in the mid-0.3 range correspond with a triage strategy that emphasizes sensitivity. The predefined probability bands (< 0.30, 0.30–0.60, ≥ 0.60) demonstrated stepwise increases in observed prevalence of PD-MCI in the training cohort (Fig. 6), supporting their face validity. It is important to note that these bands serve as triage aids rather than diagnostic cutoffs and should be tailored to local resources and risk tolerance. To promote usability, we developed a bilingual (English/Chinese) web calculator that replicates the locked coefficients and inputs of the nomogram. This tool offers standardized probability scales and band labels to improve communication between patients and healthcare teams, as well as to streamline referral workflows.

### Limitations

The labels were derived from MoCA-based MDS Level I criteria and cannot replace MDS Level II diagnosis. The tool is designed to prioritize triage and comprehensive evaluation, not to diagnose or predict long-term conversion. The development cohort comes from a single research database (PPMI), and although we tested an independent external cohort, differences in case mix and PD-MCI prevalence may affect performance metrics. The external cohort (*n* = 70) had a higher PD-MCI prevalence (∼ 41%) compared to typical epidemiological rates (∼ 20%) and was younger, which may explain the higher sensitivity observed. The small sample size of the external cohort also necessitates cautious interpretation of results. Multi-site, prospective validation using MDS Level II as the reference outcome is essential, with recalibration as needed. The model used only routinely available measures to maximize clinical deployability, though imaging and biomarkers may offer incremental value at the cost of accessibility. Finally, the cross-sectional design limits causal inference.

### Clinical implications and future work

In clinical settings where neuropsychological capacity is limited, a transparent, sensitivity-oriented, probability-based model can assist in identifying MoCA-normal patients who should receive earlier MDS Level II diagnosis and closer follow-up. Risk bands provide a straightforward method to align operational thresholds with clinic capacity: higher-sensitivity thresholds should be employed when the priority is to minimize missed cases, while more conservative thresholds are appropriate when resources are constrained. Future steps include prospective impact evaluation (e.g., time-to-MDS Level II diagnosis, appropriateness of referrals, changes in case-finding yield, patient-reported outcomes), local adaptation and monitoring of thresholds as prevalence and resources fluctuate, subgroup fairness audits (considering factors such as age, sex, and education), periodic calibration and drift surveillance, as well as the judicious integration of additional modalities (such as imaging or biomarkers) where they enhance value without compromising scalability.

## Conclusion

We developed and externally validated a probability-based risk-stratification model that used MoCA-based MDS Level I operational labels (PD-MCI vs. PD-NC) as the training outcome. The model returns a probability consistent with the MoCA-based MDS Level I PD-MCI label at the current evaluation, to help identify individuals at higher risk—particularly those with normal MoCA scores—for timely MDS Level II diagnosis and closer follow-up care. Based on comparative performance, LR was selected as the final model. For practical application, we provide prespecified probability bands and a bilingual web calculator reflecting the locked model coefficients to facilitate routine clinical workflows. The tool complements—rather than replaces—MDS diagnostic procedures and is intended for triage; it is not a diagnostic substitute nor a predictor of future conversion.

## Supplementary Information

Below is the link to the electronic supplementary material.

Supplementary Material 1

## Data Availability

Data used in the preparation of this article were obtained from the Parkinson’s Progression Markers Initiative (PPMI) database (https://www.ppmi-info.org/access-data-specimens/download-data). For up-to-date information on the study, visit https://www.ppmi-info.org. All data used in this study, as well as a data dictionary, are free and publicly available at the PPMI website. Additional, related documents including the study protocol and assay methods are also available. Data access can be requested on the website. There are no restrictions on who can request access. Find some help on our Data availability statements page.
