# Supplementary material for: Machine learning-based stratification of mild cognitive impairment in Parkinson’s disease: a multicenter cross-sectional analysis
Source: BMC Med Inform Decis Mak. 2025 Oct 15;25:384. doi: 10.1186/s12911-025-03215-0 (PMC12522980; doi:10.1186/s12911-025-03215-0)
Supplement: Supplementary file 1 — Supplementary Material 1 [file 12911_2025_3215_MOESM1_ESM.docx]

**Supplementary Material for the Manuscript:**

**Machine Learning-Based Stratification of Mild Cognitive Impairment in Parkinson’s Disease: A Multicenter Retrospective Analysis**

1. Supplementary Methods：

1.1 Nomogram Construction and Score Mapping

The nomogram was constructed based on the final logistic regression (LR) model. Each predictor’s regression coefficient (β) was transformed into a point system. This transformation was done by scaling the regression coefficient (β) by a constant factor, which was determined during the model development phase. This scaling ensures that the resulting total points correspond to clinically relevant risk levels.

Score Calculation for Each Predictor:

For each predictor variable, its regression coefficient (β) from the LR model is multiplied by a constant factor to obtain the corresponding score. The formula for calculating the points for each predictor is:Points for a predictor=β×Constant.

where β is the regression coefficient for each predictor, and Constant is a scaling factor derived during model development to ensure the score corresponds to clinically meaningful levels of risk.

Total Points: The total score for a patient is the sum of the individual scores for all predictors. This total score is then mapped to the risk of PD-MCI using a predefined mapping table, which corresponds to the model-estimated probability of PD-MCI at the current evaluation.

Mapping Total Score to Risk of PD-MCI: The total score is mapped to the risk of PD-MCI using a conversion table derived from the LR model’s output, which categorizes patients into low, intermediate, or high risk for PD-MCI based on their total score.

1. Supplementary Results：

| **Table S1.** Threshold-based performance metrics for five models at Youden-optimal cutoffs |
| --- |
| \| **character** \| **Logistic Regression** \| **SVM** \| **Neural Network** \| **XGBoost** \| **LightGBM** \| \| --- \| --- \| --- \| --- \| --- \| --- \| \| cutoff \| 0.308 \| 0.211 \| 0.306 \| 0.622 \| 0.267 \| \| AUC \| 0.789  (0.745, 0.833) \| 0.818  (0.776, 0.859) \| 0.792  (0.749, 0.834) \| 0.981  (0.970, 0.991) \| 0.908  (0.882, 0.934) \| \| Sensitivity \| 0.750  (0.670, 0.830) \| 0.893  (0.836, 0.950) \| 0.804  (0.730, 0.877) \| 1.000  (1.000, 1.000) \| 0.911  (0.858, 0.964) \| \| Specificity \| 0.694  (0.651, 0.736) \| 0.624  (0.579, 0.669) \| 0.662  (0.618, 0.706) \| 0.957  (0.939, 0.976) \| 0.761  (0.721, 0.800) \| \| Accuracy \| 0.705  (0.704, 0.706) \| 0.678  (0.677, 0.679) \| 0.691  (0.690, 0.691) \| 0.966  (0.966, 0.966) \| 0.791  (0.790, 0.791) \| \| PPV \| 0.380  (0.316, 0.444) \| 0.373  (0.315, 0.431) \| 0.373  (0.312, 0.435) \| 0.855  (0.795, 0.915) \| 0.488  (0.420, 0.556) \| \| NPV \| 0.917  (0.888, 0.947) \| 0.959  (0.936, 0.982) \| 0.931  (0.903, 0.959) \| 1.000  (1.000, 1.000) \| 0.971  (0.954, 0.989) \| \| F1 \| 0.505  (0.463,0.546) \| 0.522  (0.481,0.564) \| 0.510  (0.468, 0.551) \| 0.922  (0.900, 0.944) \| 0.636  (0.596, 0.675) \| \| Note: The Youden index was used to determine the optimal cut-off threshold for each model. The optimal cut-off point was identified where the sum of sensitivity and specificity was maximized. \| \| \| \| \| \| |

| **Table S2.** Logistic regression coefficients and odds ratios for predictors selected by LASSO |
| --- |
| \| **variable** \| **β** \| **SE** \| **z** \| **OR(95%CI)** \| ***p*** \| \| --- \| --- \| --- \| --- \| --- \| --- \| \| Sex \|  \|  \|  \|  \|  \| \| 0 \| 0.000 \|  \|  \| reference \|  \| \| 1 \| 1.074 \| 0.214 \| 5.018 \| 2.927 (1.935,4.483) \| <0.001 \| \| Education \| -1.048 \| 0.117 \| -8.995 \| 0.351 (0.277,0.438) \| <0.001 \| \| age \| 0.029 \| 0.040 \| 0.723 \| 1.029 (0.948,1.104) \| 0.470 \| \| Ageonset \| 0.028 \| 0.039 \| 0.711 \| 1.028 (0.961,1.115) \| 0.477 \| \| Upsit \| -0.036 \| 0.014 \| -2.484 \| 0.965 (0.938,0.992) \| 0.013 \| \| Gds \| 0.124 \| 0.037 \| 3.397 \| 1.132 (1.054,1.217) \| 0.001 \| \| Updrs3_score \| 0.105 \| 0.011 \| 9.164 \| 1.111 (1.087,1.137) \| <0.001 \| \| Note: These values correspond to the LR model selected at the 1-SE LASSO criterion. \| \| \| \| \| \| |

| Figure S1. Example of nomogram application for a high-risk patient. |
| --- |
| 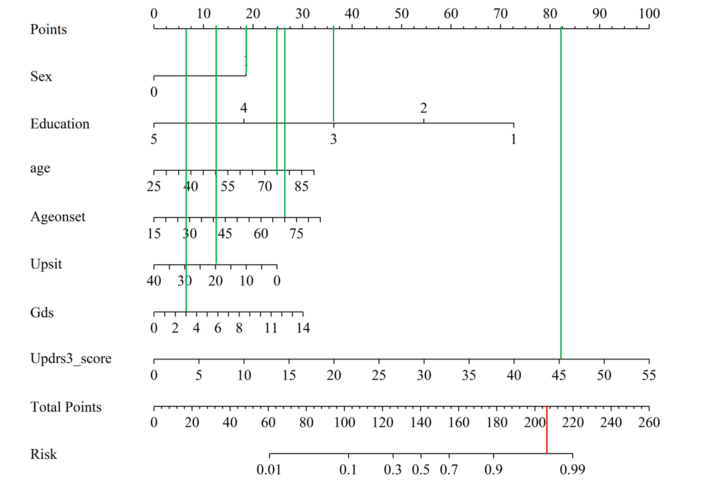 |
| Note: Green lines indicate the points assigned to each predictor according to its value. The red line represents the total points and the corresponding predicted probability of PD-MCI. A 75-year-old male (Education=3, Age at onset=70, UPDRS III=45, GDS=3, UPSIT=20) scores 206 total points, corresponding to the risk of PD-MCI probability of 0.97. |

| **Figure S2.** Screenshots of the online risk calculator for high-, intermediate-, and low-risk patients |
| --- |
| 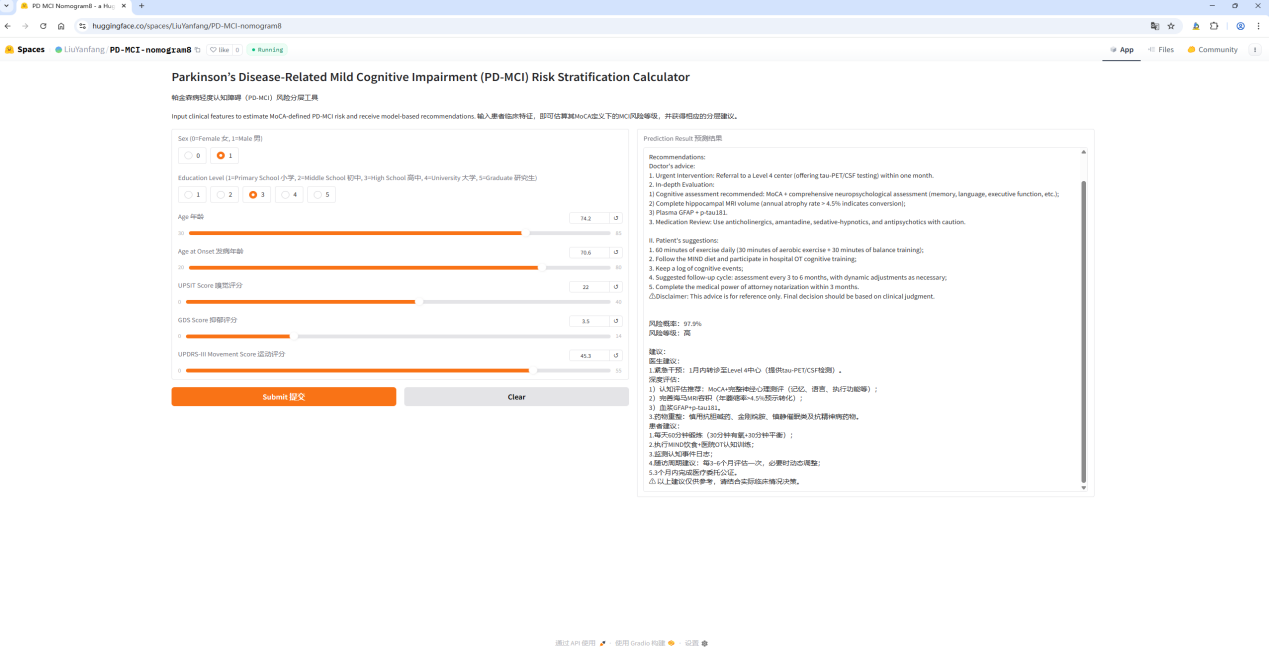 |
| 1. High-risk patient |
| 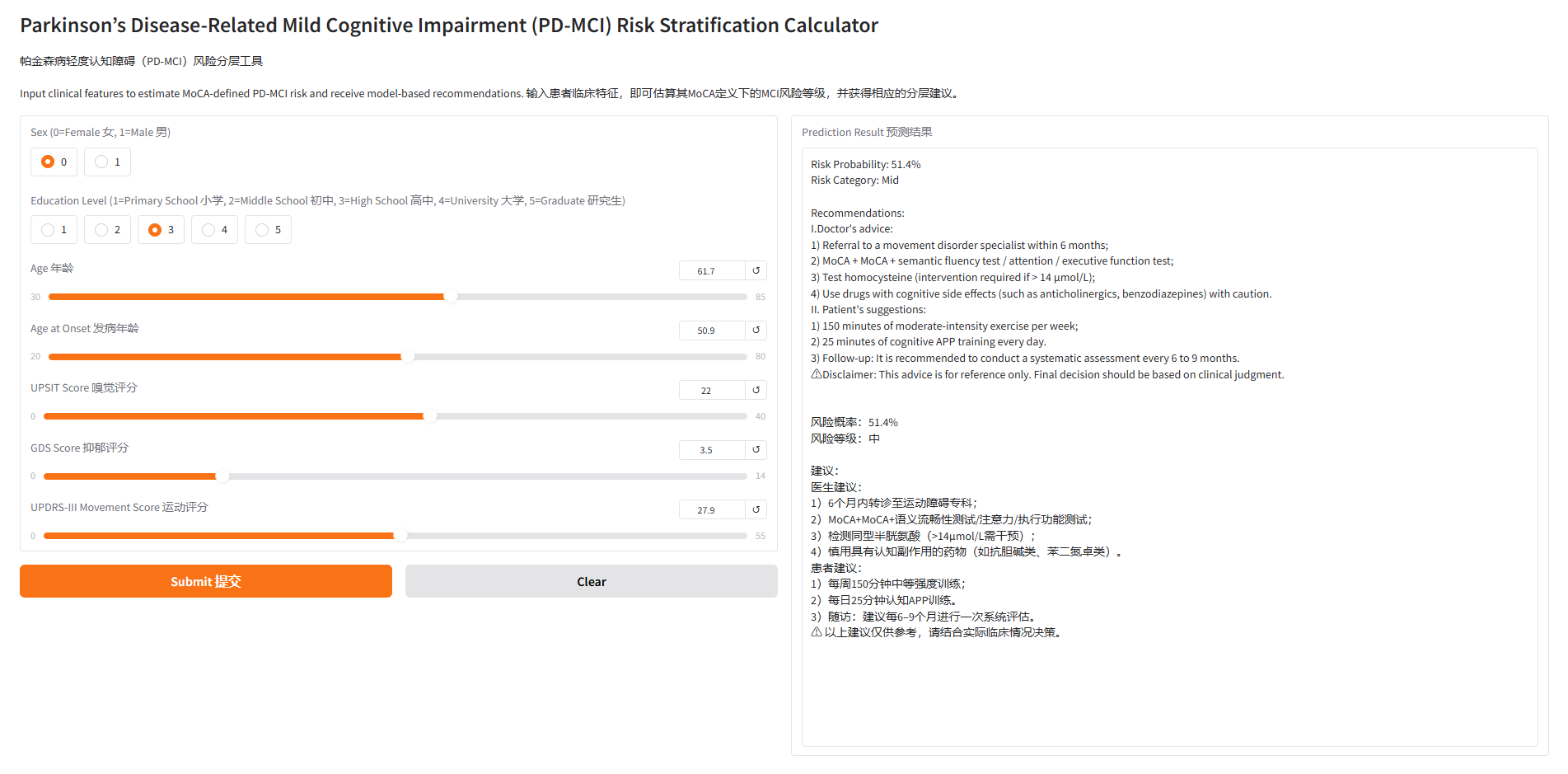 |
| 1. Intermediate-risk patient |
| 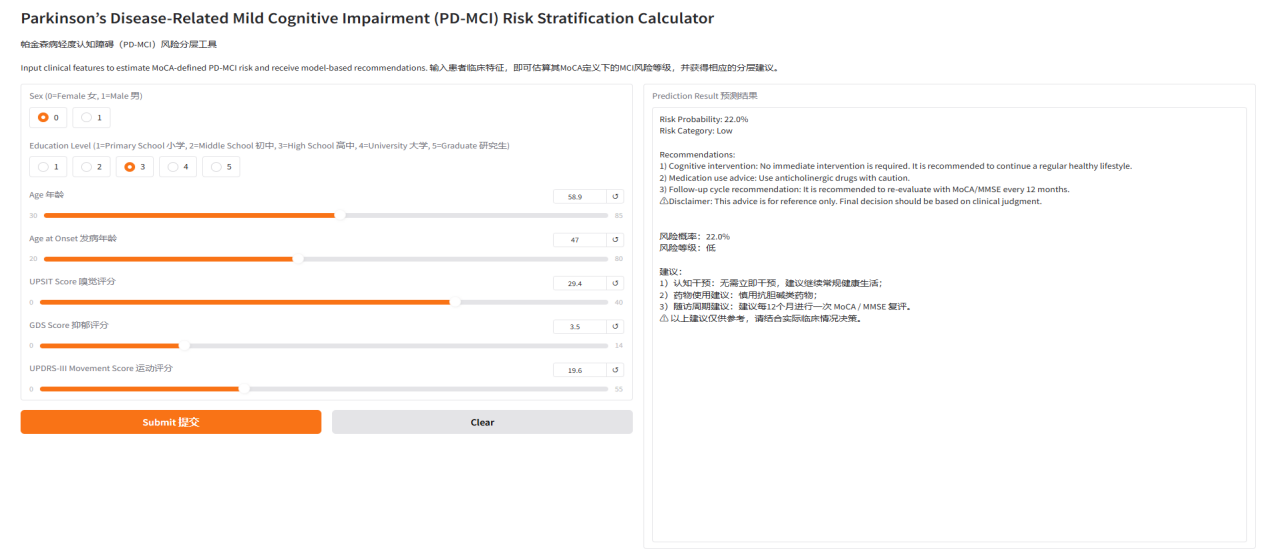 |
| 1. Low-risk patient |
